# The complete mitochondrial genome of *Fieberiella septentrionalis* (Hemiptera: Cicadellidae: Deltocephalinae)

**DOI:** 10.1080/23802359.2021.1912671

**Published:** 2021-04-15

**Authors:** Huan Luo, Yuan Wang, Xiao-chen Di, Liang-chen-yu Shan, Shao-shan Wang

**Affiliations:** a Agricultural College of Shihezi University, Key Laboratory of Oasis Agricultural Pest Management and Plant Protection Resources Utilization, Shihezi, Xinjiang, China; b Shaanxi International Travel Healthcare Center, Xi'an Customs, Xi'an, China; c College of Life Sciences & Technology, Inner Mongolia Normal University, Hohhot, China

**Keywords:** Deltocephalinae, mitogenome phylogeny, *Fieberiella septentrionalis*

## Abstract

The complete mitochondrial genome of *Fieberiella septentrionalis* was annotated for the first time in the present study. The mitogenome was found to have circular shape, with 16,175 bp in size, containing 13 protein coding genes (PCGs), 22 transfer-RNA genes, 2 ribosomal-RNA genes, and 1 non-coding region. The nucleotide composition biases toward A and T is 77.9% of the entirety, which is a typical structure of Cicadellidae. All PCGs have ATN as the start codon, TAA and single T as the stop codon. The resulting phylogenetic tree confirms that the *F. septentrionalis* belongs to the subfamily of Deltocephalinae and Fieberiellini as sister to the remaining tribes of this subfamily.

Deltocephalinae is the largest and most economically important subfamily of leafhoppers, presenting distinct diagnostic characteristics and including over 6600 described extant species and 39 tribes (Zahniser and Dietrich 2013). Among the phylogenetic studies of the group, the taxon and character sampling in the morphological analysis of Deltocephalinae by Zahniser and Dietrich (2008) is the most comprehensive to date. *Fieberiella septentrionalis* Wagner (1963) belongs to Fieberiellini of the subfamily. It is distributed in Palearctic, Nearctic, and African, and often parasitizes dicotyledon trees and shrubs. It was first discovered by Wanger (1963) in Germany, but there is no information about the collection site and phylogenetic relationship in their article. The complete mitochondrial genome of *F. septentrionalis* reported in this article is for further study of its taxonomy and constructed phylogenetic tree to further validate taxonomic status on genome level.

In this study, we sequenced and annotated the complete mitochondrial DNA of the *F. septentrionalis* for the first time. The specimen of *F. septentrionalis* was collected in Shihezi city, Xinjiang Uygur Autonomous Region, China (N44.3122, E86.0569), in September 2019. Fresh specimens (voucher number IMNU20190908) were initially preserved in 100% ethanol, and then stored at −20 °C in the laboratory. After morphological identification, the entire body without abdomen was shipped to Tsingke (Beijing, China) for genomic extraction. The qualified genomic DNA is fragmented by mechanical interruption (ultrasonic), then the fragment is purified, end repaired, connected to the sequencing adapter, and selected by agarose gel electrophoresis. The library with insert size of 300 bp fragments was constructed using the PCR amplification and then sequenced was performed on the Illumina HiSeq 2000 instrument. The number and quality of raw paired-end reads were evaluated by using the FastQC (Andrews 2018). *De novo* assembly of clean reads was performed using SPAdes v3.11.0 (Bankevich et al. 2012). The mitogenome of *Macrosteles quadrilineatus* (GenBank accession number KY645960) was further used as a reference to assemble the sequenced sample. The mitochondrial genome was annotated with Geneious 9.1.4. All 13 protein-coding genes and 2 rRNA genes were determined by comparison with the homologous sequences of other leafhoppers from GenBank. The 22 tRNA genes were identified by MITOS WebServer (Bernt et al. 2013). The annotated sequence of *F. septentrionalis* mitogenome was deposited in GenBank with an accession number MW078430.

The mitochondrial genome of *F. septentrionalis* is 16,175 bp in length, with the A + T content of 77.9% (T 35.4%, C 12.6%, A 42.5%, and G 9.5%), which is a typical structure of Cicadellidae mitogenome (74–85%; Chen et al. 2020). Annotation of the mitogenome revealed 13 protein-coding genes (PCGs) (COX1-3, ND1-6, ND4L, ATP6, ATP8, and Cytb), 22 transfer-RNA (tRNA) genes, two ribosomal RNA unit genes (rRNAs) and one A-T rich region (Control region). Its gene arrangement and direction are in accordance with other Cicadellidae leafhopper (Boore 1999). Across the 13 PCGs in *F. septentrionalis*, only four genes (ND4, ND4L, ND5, and ND1) are coded on the minority strand (N-strand), whereas the others are coded on the majority strand (J-strand). Generally, PCGs are started with ATN codon and terminate with the stop codons TAA and TAG except for COX2 ended with single T. The 16S rRNA gene is 1224 bp in length and is located between tRNA-L1 and tRNA-V; the 12S rRNA gene is 755 bp in size and is located between tRNA-V and A-T-rich region. The A-T region is 1727 bp in size and located after the 12S rRNA.

Here, we reconstructed a phylogeny of Cicadellidae using 13 PCGs from *F. septentrionalis* and other 24 representatives from 5 subfamilies of the family and 2 outgroup species from Cercopoidea and Cicadoidea (Figure 1). Thirteen concatenated PCG sequences of mitogenomes were analyzed by the Bayesian inference (BI) method in MrBayes 3.2.6 (Ronquist et al. 2012). The optimal partitioning scheme and nucleotide substitution model for Bayesian inference (BI) phylogenetic analyses based on the nucleotide dataset of 13 PCGs were selected with PartitionFinder 2.1.1 (Lanfear et al. 2016) incorporated into PhyloSuite v1.2.1, using the branch lengths linked, Bayesian information criterion (BIC) model and the greedy search algorithm (Lanfear et al. 2012). The phylogenetic tree showed that Deltocephalinae was a monophyletic group in Cicadellidae and Fieberiellini was sister of the remaining tribes of this subfamily.

**Figure 1.**
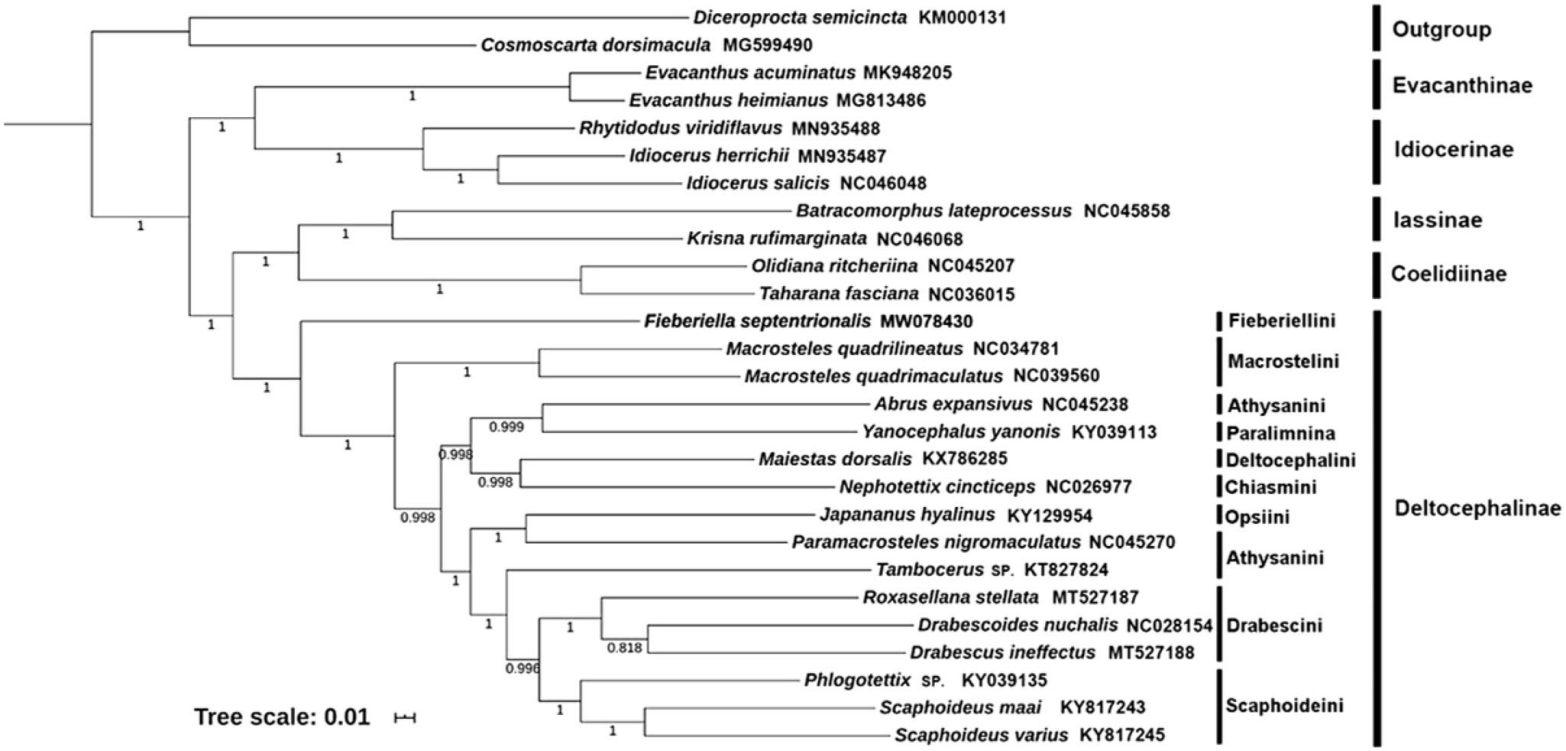
Phylogenetic tree inferred from BI method based on the nucleotide dataset of 13 PCGs. Numbers on branches are posterior probabilities (PP).

## Data Availability

The genome sequence data that support the findings of this study are openly available in GenBank of NCBI at https://www.ncbi.nlm.nih.gov/ under the Accession no. MW078430. The associated BioProject, SRA, and Bio-Sample numbers are PRJNA705294, SRP13838339, and SAMN18080047, respectively.
